# Supplementary material for: Working memory control dynamics follow principles of spatial computing
Source: Nat Commun. 2023 Mar 14;14:1429. doi: 10.1038/s41467-023-36555-4 (PMC10015009; doi:10.1038/s41467-023-36555-4)
Supplement: Supplementary file 1 — Supplementary Information [file 41467_2023_36555_MOESM1_ESM.pdf]

Supplemental Figures

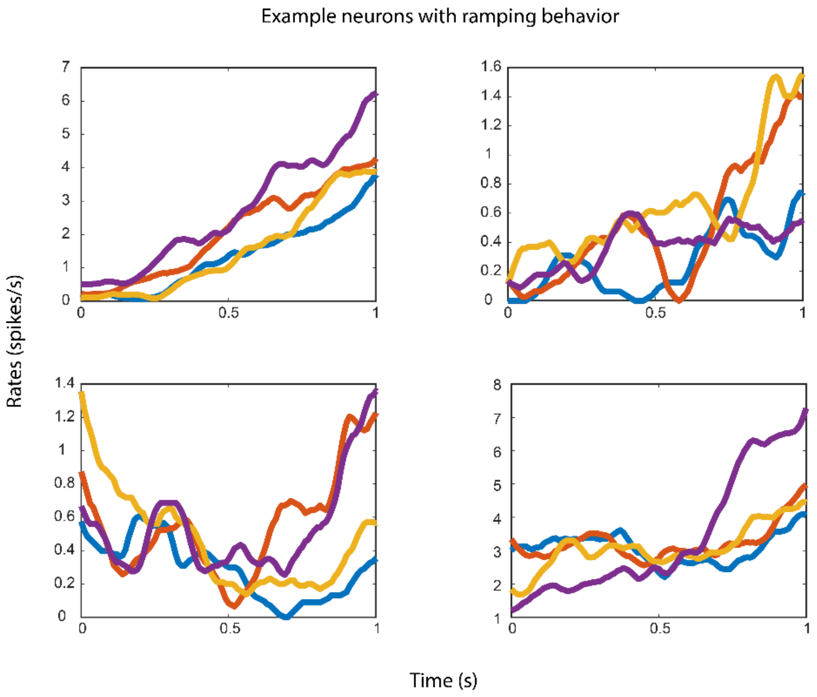

Figure S1. Single neuron examples of ramping activity. Shown are the activity grouped by sample identity (blue/red/yellow/purple) for all four neurons with ramping activity from two random sessions.

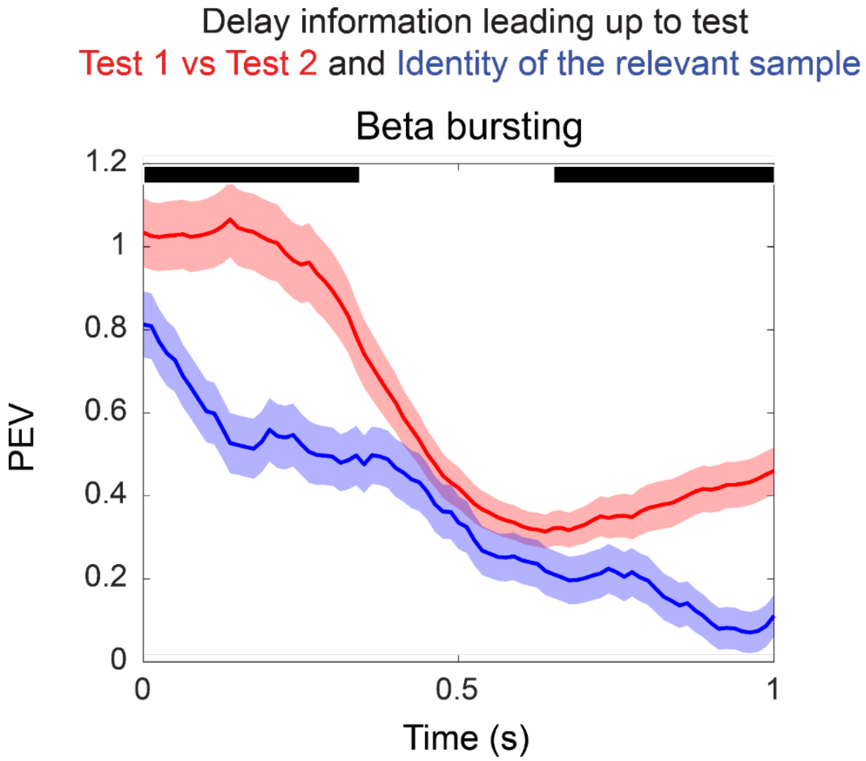

Figure S2. *Beta information about sample and upcoming test. Red plots show PEV accounting for test order effects estimated over two groups of periods, preceding either Test 1 or Test 2. Blue curves reflect PEV wrt. sample identity (information about identity about Sample 1 prior to Test 1 and Sample 2 leading up to Test 2). Black bars demonstrate when blue and red plots differ, using cluster based statistics (p<0.05, n=199). Shaded regions represent SEM.*

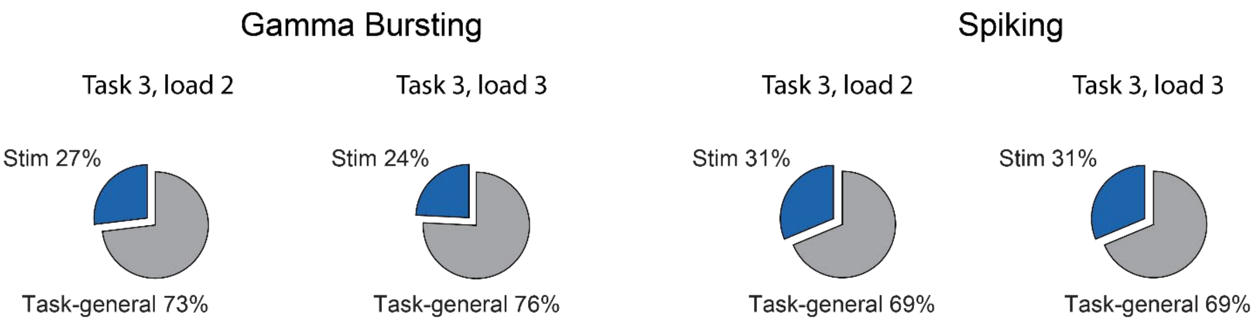

Figure S3. dPCA analysis of Task 3. Shown are the proportion of variance that can be attributed to stimulus (blue) and condition-independent (grey) components for gamma (left) and spiking (right). Due to their distinct task structures, load 2 and load 3 were analyzed separately.

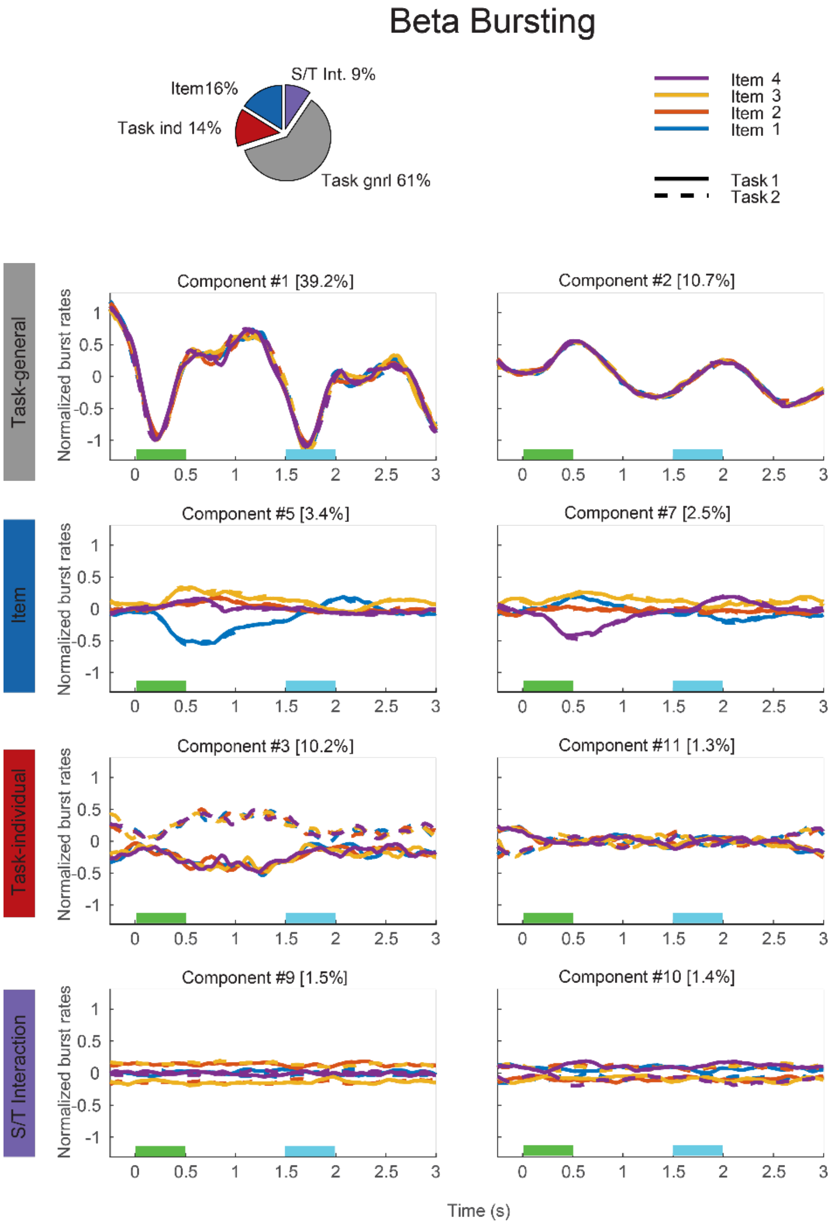

Figure S4. Demixed principal component analysis (dPCA) of beta. *Here we used dPCA [20] to extract the principal components and attribute them to task control-related and item-specific activity. "Task general" (grey) and "Task individual" (red) components correspond to low-dimensional task control-related activity (n=199). "Task general" components reflect shared patterns of activity over time in the two tasks whereas "Task individual" components explain the variance that originates from the differences between the two tasks. Item dependent components (blue) account for the variance between four different cued items (item-specific activity).* The green (sample 1) and light blue (sample 2) rectangles marks when the samples where shown. "S/T int" refers to components that depended both on item and task when data from both tasks where analyzed together. The bottom half of the figure shows example components for Task 1 and Task 2 combined. Shown are the first two components of each type (meaning several task general components are not shown as they tended to dominate).

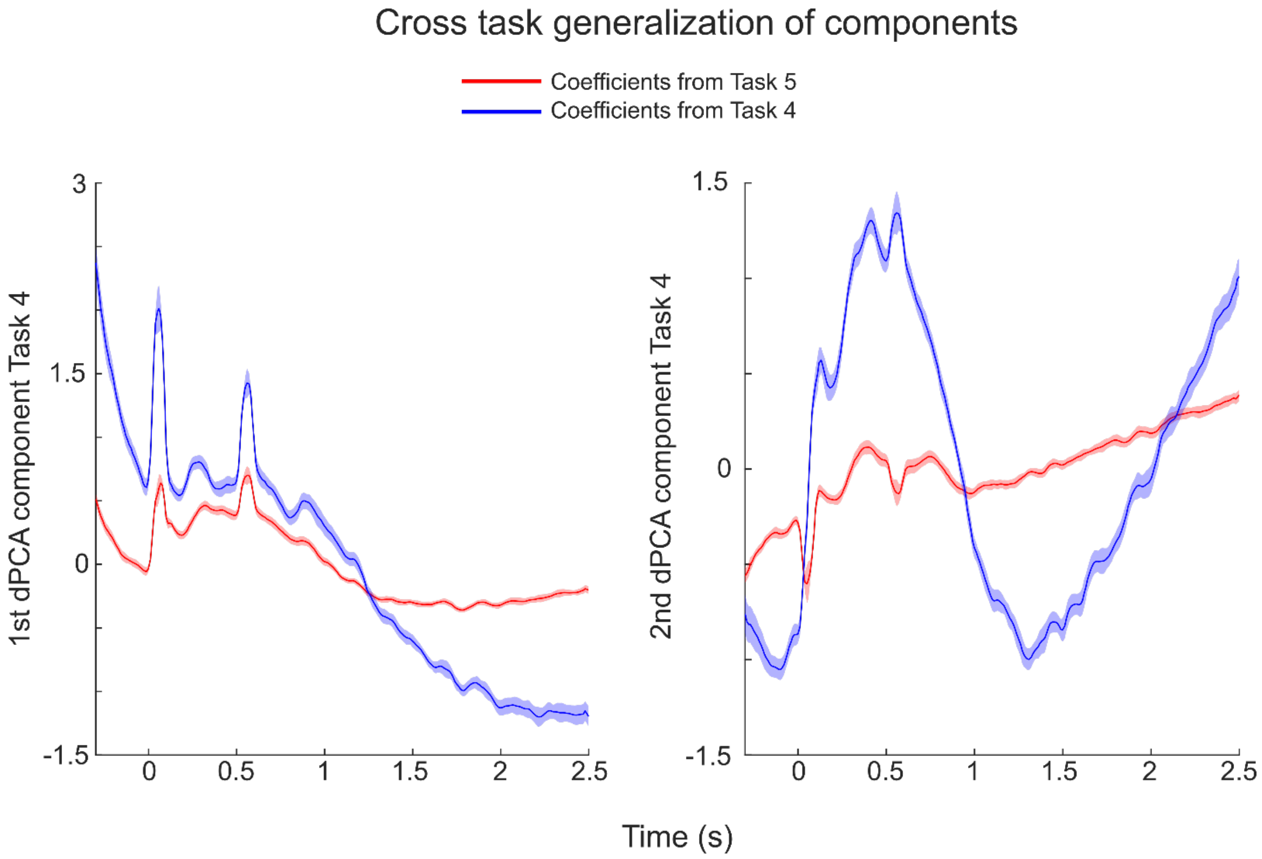

Figure S5. *Cross-task generalization of spatial distribution of components. Left: Plotted is data (5 sessions, 232 electrodes each) from Task 4 projected using coefficients from the first control-related component extracted from Task 4 (blue) and Task 5 (red). Right: Same as "Left" but for the second task-related component instead. Shaded regions denote SEM across sessions. The difference between red and blue curves suggests the components for the two tasks are only partly overlapping spatially.*

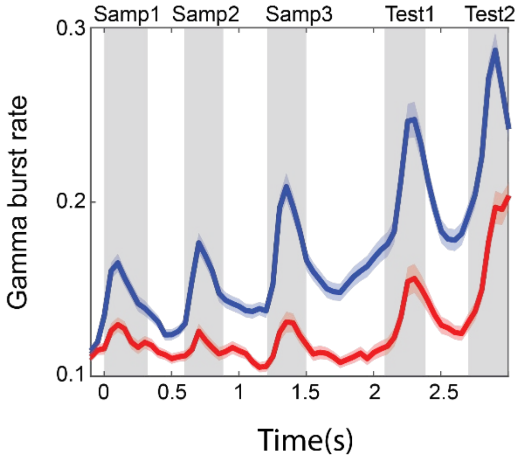

Figure S6. *Gamma bursting reflects the excitation in the network. Blue curves (n=64) correspond to sites in which neurons are selective to items cued at Samples 1-3, red curves describe sites with no such selective neurons (n=198). Trials with three sequential sample cues and two test cues (monkeys correctly respond at Test 2) are shown. Shaded regions denote SEM across recording sites.*
