# Supplementary material for: Working memory control dynamics follow principles of spatial computing
Source: Nat Commun. 2023 Mar 14;14:1429. doi: 10.1038/s41467-023-36555-4 (PMC10015009; doi:10.1038/s41467-023-36555-4)
Supplement: Supplementary file 2 — Reporting Summary [file 41467_2023_36555_MOESM2_ESM.pdf]

nature portfolio

Corresponding author(s): Pawel Herman, Mikael Lundqvist

Last updated by author(s): 16/12/22

Reporting Summary

Nature Portfolio wishes to improve the reproducibility of the work that we publish. This form provides structure for consistency and transparency in reporting. For further information on Nature Portfolio policies, see our Editorial Policies and the Editorial Policy Checklist.

Statistics

For all statistical analyses, confirm that the following items are present in the figure legend, table legend, main text, or Methods section.

n/a	Confirmed	
☐	☒	The exact sample size (*n*) for each experimental group/condition, given as a discrete number and unit of measurement
☐	☒	A statement on whether measurements were taken from distinct samples or whether the same sample was measured repeatedly
☐	☒	The statistical test(s) used AND whether they are one- or two-sided<br>*Only common tests should be described solely by name; describe more complex techniques in the Methods section.*
☒	☐	A description of all covariates tested
☐	☒	A description of any assumptions or corrections, such as tests of normality and adjustment for multiple comparisons
☐	☒	A full description of the statistical parameters including central tendency (e.g. means) or other basic estimates (e.g. regression coefficient) AND variation (e.g. standard deviation) or associated estimates of uncertainty (e.g. confidence intervals)
☐	☒	For null hypothesis testing, the test statistic (e.g. *F*, *t*, *r*) with confidence intervals, effect sizes, degrees of freedom and *P* value noted<br>*Give P values as exact values whenever suitable.*
☒	☐	For Bayesian analysis, information on the choice of priors and Markov chain Monte Carlo settings
☒	☐	For hierarchical and complex designs, identification of the appropriate level for tests and full reporting of outcomes
☒	☐	Estimates of effect sizes (e.g. Cohen's *d*, Pearson's *r*), indicating how they were calculated

Our web collection on statistics for biologists contains articles on many of the points above.

Software and code

Policy information about availability of computer code

Data collection	MonkeyLogic was used to collect behavioural data. Electrophysiological data were collected using Blackrock Cerebus ver. 2.3 with the support of NPMK software ver. 2.6.0.0.
Data analysis	Key functions for estimating a percentage of explained variance (PEV) and oscillatory burst analysis are available online (https://kth-my.sharepoint.com/:f:/g/personal/paherman_ug_kth_se/EtzuzXGiGGZOoloNoOBse34BPbQTmgYQA5AnoMBDby8SuQ?e=2Vklun). External tools such as Fieldtrip toolbox (Oostenveld et al., 2011; v20190422, https://www.fieldtriptoolbox.org/) employed for spectral analysis and dPCA toolbox (Kobak et al., 2016; ver. July 15, 2016, https://github.com/machenslab/elife2016dpca) are published online. Data analysis was performed using Matlab R2019a (The Mathworks, Natick, MA, USA).

For manuscripts utilizing custom algorithms or software that are central to the research but not yet described in published literature, software must be made available to editors and reviewers. We strongly encourage code deposition in a community repository (e.g. GitHub). See the Nature Portfolio guidelines for submitting code & software for further information.

Data

Policy information about availability of data

All manuscripts must include a data availability statement. This statement should provide the following information, where applicable:

- Accession codes, unique identifiers, or web links for publicly available datasets
- A description of any restrictions on data availability
- For clinical datasets or third party data, please ensure that the statement adheres to our policy

The data that support the findings of this study are available from one of the authors (E.K.M.) upon reasonable request. Source data are published online with the paper.

Human research participants

Policy information about studies involving human research participants and Sex and Gender in Research.

Reporting on sex and gender	N/A
Population characteristics	N/A
Recruitment	N/A
Ethics oversight	N/A

Note that full information on the approval of the study protocol must also be provided in the manuscript.

Field-specific reporting

Please select the one below that is the best fit for your research. If you are not sure, read the appropriate sections before making your selection.

☒ Life sciences ☐ Behavioural & social sciences ☐ Ecological, evolutionary & environmental sciences

For a reference copy of the document with all sections, see nature.com/documents/nr-reporting-summary-flat.pdf

Life sciences study design

All studies must disclose on these points even when the disclosure is negative.

Sample size	No explicit power analysis was made when the study was designed. Most data used in the research had been published before. Thus the maximum available sample size was exploited. As for the new data source, we only included the largest subset that allowed a cross-session comparison. The rest of the data contained variations in timings and other aspects of the experimental design. Again, we exploited the maximum available sample size.
Data exclusions	All available data was exploited to maximise the sample size given constraints regarding the signal quality (all explained in Methods). In particular, only electrodes containing isolatable units were kept for further analysis and electrodes displaying high noise were considered as outliers and removed (explained in the Signal Processing subsection of Methods).<br>Also, the exclusion criterion was adopted for analysis reported in Fig.6B and Fig.6C, where data from two recording sessions were excluded due to the lack of correct trials for one of the task conditions, which would have introduced bias in the estimate of PEV information. This is mentioned in Fig.6 caption.<br>The criteria for categorizing/grouping units or electrodes as informative/selective are clearly described in the Results section.<br>The only arbitrary selection of a data subset was made to illustrate examples in Fig.S1, where two consecutive recording sessions were randomly picked for presentation purposes. The selection was made based on visual inspection though all data demonstrated coherent trends.
Replication	The experiments were performed by the same researchers over several sessions. No other replication of the experimental process was made. Therefore the only replication in this study is concerned with data analysis. Most of analyses were first performed on a data subset before full scale analysis was conducted. A number of control analyses with a range of manipulations were also implemented to validate the results.
Randomization	Experimental trials were randomized and balanced between conditions of interest.
Blinding	Thousands of trials for each condition were collected from the animals and analysis was applied to a large data set, which the researchers were blind to.

Reporting for specific materials, systems and methods

We require information from authors about some types of materials, experimental systems and methods used in many studies. Here, indicate whether each material, system or method listed is relevant to your study. If you are not sure if a list item applies to your research, read the appropriate section before selecting a response.

Materials & experimental systems

n/a	Involved in the study
☒	☐ Antibodies
☒	☐ Eukaryotic cell lines
☒	☐ Palaeontology and archaeology
☐	☒ Animals and other organisms
☒	☐ Clinical data
☒	☐ Dual use research of concern

Methods

n/a	Involved in the study
☒	☐ ChIP-seq
☒	☐ Flow cytometry
☒	☐ MRI-based neuroimaging

Animals and other research organisms

Policy information about studies involving animals; ARRIVE guidelines recommended for reporting animal research, and Sex and Gender in Research

Laboratory animals	In total 5 rhesus macaques, Macaca mulatta (4 males and 1 female), between the ages of 5 and 12 were involved in the study.
Wild animals	No wild animals were used in the study.
Reporting on sex	N/A
Field-collected samples	No field collected studies were used in the study.
Ethics oversight	Massachusetts Institute of Technology Committee on Animal Care

Note that full information on the approval of the study protocol must also be provided in the manuscript.
